# Liver Fibrosis in Type I Gaucher Disease: Magnetic Resonance Imaging, Transient Elastography and Parameters of Iron Storage

**DOI:** 10.1371/journal.pone.0057507

**Published:** 2013-03-15

**Authors:** Anneloes E. Bohte, Laura van Dussen, Erik M. Akkerman, Aart J. Nederveen, Ralph Sinkus, Peter L. M. Jansen, Jaap Stoker, Carla E. M. Hollak

**Affiliations:** 1 Department of Radiology, Academic Medical Center, University of Amsterdam, Amsterdam, The Netherlands; 2 Department of Endocrinology and Metabolism, Academic Medical Center, University of Amsterdam, Amsterdam, The Netherlands; 3 CRB3, UMR 773, Inserm, Université Paris Diderot, Sorbonne Paris Cité, Clichy, France; 4 Department of Gastroenterology and Hepatology, Academic Medical Center, University of Amsterdam, Amsterdam, The Netherlands; University Hospital S. Maria della Misericordia, Italy

## Abstract

Long term liver-related complications of type-1 Gaucher disease (GD), a lysosomal storage disorder, include fibrosis and an increased incidence of hepatocellular carcinoma. Splenectomy has been implicated as a risk factor for the development of liver pathology in GD. High ferritin concentrations are a feature of GD and iron storage in Gaucher cells has been described, but iron storage in the liver in relation to liver fibrosis has not been studied. Alternatively, iron storage in GD may be the result of iron supplementation therapy or regular blood transfusions in patients with severe cytopenia. In this pilot study, comprising 14 type-1 GD patients (7 splenectomized, 7 non-splenectomized) and 7 healthy controls, we demonstrate that liver stiffness values, measured by Transient Elastography and MR-Elastography, are significantly higher in splenectomized GD patients when compared with non-splenectomized GD patients (p = 0.03 and p = 0.01, respectively). Liver iron concentration was elevated (>60±30 µmol/g) in 4 GD patients of whom 3 were splenectomized. No relationship was found between liver stiffness and liver iron concentration. HFE gene mutations were more frequent in splenectomized (6/7) than in non-splenectomized (2/7) participants (p = 0.10). Liver disease appeared more advanced in splenectomized than in non-splenectomized patients. We hypothesize a relationship with excessive hepatic iron accumulation in splenectomized patients. We recommend that all splenectomized patients, especially those with evidence of substantial liver fibrosis undergo regular screening for HCC, according to current guidelines.

## Introduction

Gaucher disease (GD) is one of the most prevalent lysosomal storage disorders, currently treatable with enzyme replacement therapy (ERT) and substrate reduction therapy (SRT) [1]–[5]. Long term liver complications that have been associated with GD include fibrosis and cirrhosis [6]. In addition, several studies have reported on the occurrence of hepatocellular carcinoma (HCC) among GD patients [7]–[13] and one cohort study reported that the risk of developing HCC was increased in GD patients, suggesting that splenectomy – formerly the only treatment option – could be a risk factor for the development of HCC in GD patients as a result of advanced hepatic involvement after splenectomy [8]. The pathophysiology, however, has not been fully elucidated. Immunological abnormalities such as T-cell dysfunction or chronic stimulation of the immune system have been implicated in the pathophysiology of the elevated cancer risk in GD patients [14], [15]. An alternative hypothesis is the relationship with increased iron storage in the liver, in which iron promotes the formation of reactive oxygen species, causing hepatocellular injury. Raised serum ferritin concentrations are a well-known laboratory feature of GD, and can be used to monitor response to treatment [16]–[21]. These high ferritin concentrations have been suggested to reflect an altered iron metabolism in GD [22].

Both ERT and SRT have been shown to be highly effective in reversing cytopenia and reducing organ volumes. Splenectomies are now rarely necessary [23]–[25]. However, a substantial number of GD patients currently on treatment have had a splenectomy in the past. These patients may suffer from residual disease, which may cause partial unresponsiveness to treatment [26]–[28]. As this cohort ages, particular care should be taken to ensure proper monitoring with respect to the occurrence of long term complications, including HCC. Since liver fibrosis and hepatic iron storage are known risk factor for the development of HCC, screening for both entities could therefore be of additional value in the management of GD patients [29], [30].

Liver biopsy is not a desirable procedure to evaluate the presence of fibrosis and iron accumulation in GD, given the patients’ increased bleeding tendency. Lately however, new non-invasive and accurate quantitative imaging methods have become available by which a number of liver parameters can be evaluated. Transient Elastography (TE) and magnetic resonance elastography (MRE) both measure tissue stiffness of the liver, which is associated with the presence of fibrosis [31]–[33]. The techniques can predict the presence of cirrhosis and related complications such as portal hypertension and both techniques have shown to be of value for the non-invasive detection of liver fibrosis in patients with chronic liver disease [34], [35]. Liver iron concentration (LIC) can also be measured non-invasively using MRI, as iron shortens T1, T2, and T2^*^ relaxation times, leading to a lower signal intensity of the hepatic parenchyma, representing total body iron stores [36]–[38].

We speculate that the increased HCC risk in splenectomized GD patients is related to the presence of fibrosis and iron. Our objective therefore was to investigate whether GD patients with and without splenectomy (Sx) differed with respect to liver stiffness and iron content.

## Materials and Methods

### Ethics Statement

This study was approved by the institutional review board of the Academic Medical Center, Amsterdam, The Netherlands. All clinical investigations were conducted according to the principles expressed in the Declaration of Helsinki. Written informed consent was obtained from all participants.

### Study Design and Participants

Between April 2010 and March 2012 we prospectively included 23 adult participants: eight splenectomized (Sx) GD patients, eight non-splenectomized (non-Sx) GD patients and seven healthy controls, matched for age and sex with the non-Sx GD group.

GD patients were eligible if they had been on treatment for at least two years (either ERT or SRT). Exclusion criteria for all participants were: known chronic liver disease (viral hepatitis, HIV, primary biliary cirrhosis, primary sclerosing cholangitis, autoimmune hepatitis, Wilson’s disease, a-1-antitripsinedeficiency, abetalipoproteinemia), type 2 diabetes, use of drugs with known steatogenic effects on the liver (with the exception of oral contraceptives), alcohol consumption of >3 units/day for males and >2 units/day for females or contra-indications for MRI. History of iron intake and blood transfusion were recorded for all GD patients.

### Study Procedures

**Figure 1:**
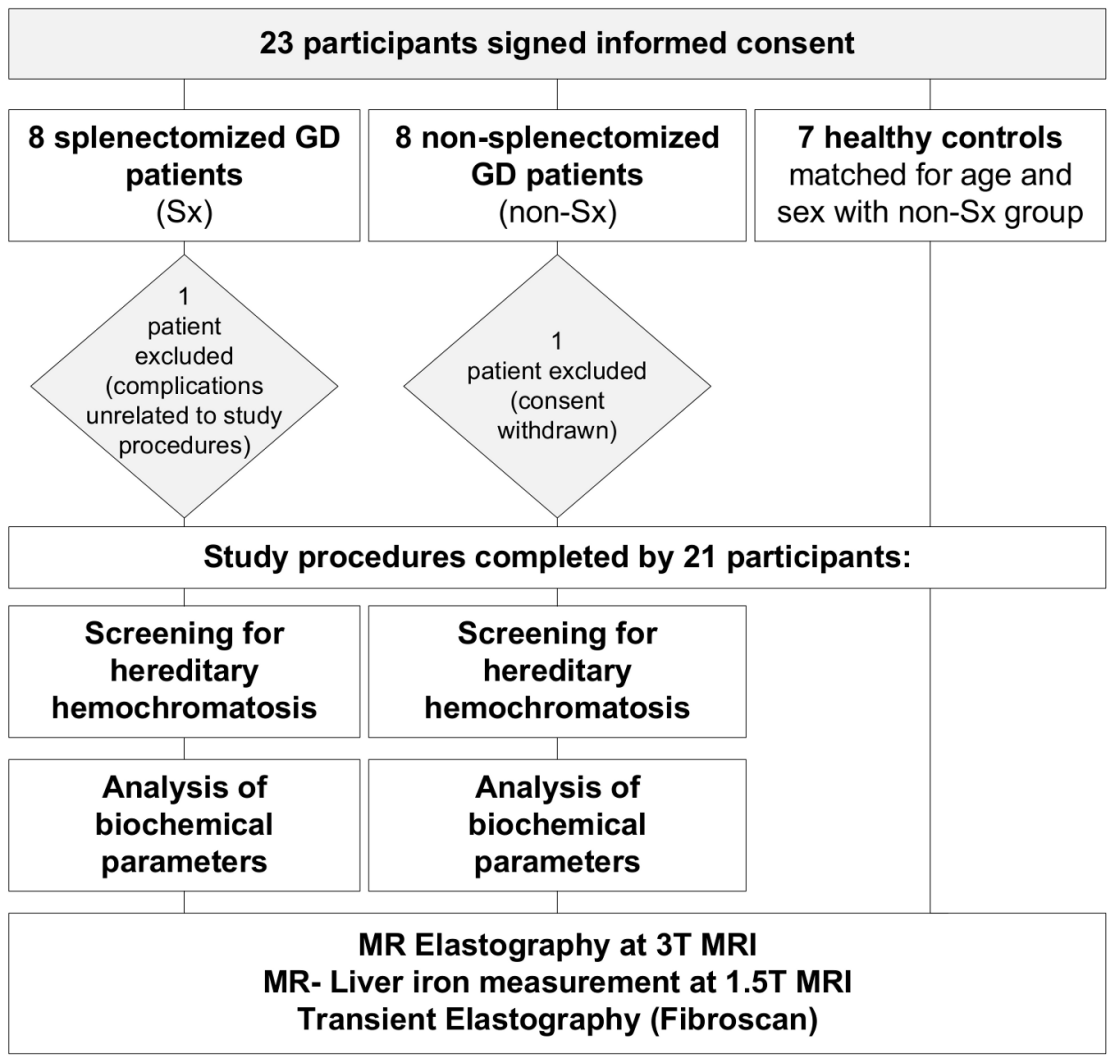
Study flow chart.

A flow-chart of the study procedures is shown in Figure 1. Most GD patients visit the outpatient clinic two to four times a year. All study procedures were therefore performed on the same day as the scheduled regular appointment. If we were unable to complete all procedures on this single day, this was done at the next scheduled appointment.

### Quantitative Imaging (All Participants)

#### Liver Iron Concentration (LIC)

LIC measurements were performed at 1.5 Tesla MR system (Siemens, Magnetom Avanto, Germany). Two accepted methods were applied in all participants during a single MR session:

1. LIC measurement according to Gandon et al. [39], in which an online quantification algorithm was used (http://www.radio.univ-rennes1.fr). Signal intensity values were measured manually in regions of interest (ROI) in the liver and paraspinous muscle on five scans, which were each acquired using a gradient echo sequence with varying echo times and flip angles (FA), resulting in T1, proton density and increasing T2 weighting. The Gandon method consisted of 5 breath hold scans of 20 seconds each. Signal intensity values were entered online, and a mean LIC ±95% confidence interval (CI) in µmol/g was returned. A LIC value is significantly elevated when the lower limit of the returned 95% CI equals 36 µmol/g or above. This corresponds to a mean LIC value of approximately >60 µmol/g, as the magnitude of the 95% CIs is usually ±20 µmol/g or greater [39].
2. T2* measurement: Gradient echo sequence with repetition time 300 ms, 12 echoes with echo times from 0.99 to 16.5 ms in steps of 1.41 ms, number of slices 3, slice thickness 10 mm, voxel size 3×3 mm, FA 20° and field of view 380×380 mm. T2* relaxation times, which are inversely proportional to LIC, were calculated from the obtained images. T2* anatomical maps were created for both the liver and, if present, the spleen, giving an impression of the heterogeneity of iron distribution. Reference values for T2* relaxation times were derived from St. Pierre et al: approximately >22 ms [36], [37]. The T2* acquisition comprised a single breath hold scan of 15 seconds.

#### Transient Elastography (TE)

TE (Fibroscan^©^, Echosens, France) measures the propagation speed of a shear wave using ultrasound. The shear waves are generated by a small vibrating device that is positioned on the top of the ultrasound transducer. The velocity of the propagating shear waves correlates with tissue stiffness. TE can measure liver stiffness in a cylinder of approximately 1 cm in diameter and 4 cm in length [40].

TE was performed by three examiners, one expert (>500 measurements) and two less experienced examiners (<100 measurements), who were trained by the expert. Measurements were carried out at three different locations, taking into account the often heterogeneous liver morphology in GD patients.

1. At the intersection between the xyphoid line and the median axillary line (reference location).
2. 2–3 cm anterior of the reference position in the same intercostal space (anterior location).
3. 2–3 cm posterior to the reference position in the next intercostal space on the same xyphoid line as the reference position (posterior location).

A minimum of 10 measurements were performed. Median liver stiffness (mLS), success rate (SR) and interquartile range (IQR) was recorded. The following reliability criteria for TE were applied [41]:

very reliable: IQR/mLS ≤0.10;
reliable: IQR/mLS between 0.10 and 0.30, or IQR/mLS >0.30 with mLS <7.1 kPa;
poorly reliable: IQR/mLS >0.30 with mLS ≥7.1 kPa

#### MR Elastography (MRE)

**Figure 2:**
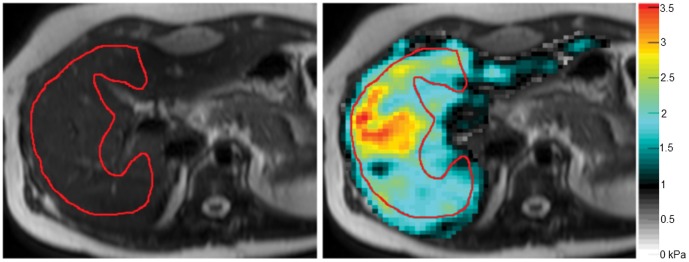
MRE: selection of region of interest (ROI) in the liver. ROIs are drawn manually on the elastograms, in the lateral part of the right liver lobe while avoiding liver margins and large hepatic vessels. The mean elasticity value within the ROI in this example (Sx GD patient) is 2.3 kPa (colored bar represents kPa). The corresponding stiffness measured with TE was 7.2 kPa.

The principle of MRE is comparable to that of TE. Low frequency mechanical waves are sent into the liver by a portable transducer, inducing shear stresses while propagating. A motion-sensitive MRI sequence measures the resulting displacement fields (Figure 2 B), in three orthogonal directions. From these 3D- displacement fields, the shear modulus G* is analyzed. The real part of G* is the *storage modulus G′*. This storage modulus G′ can be referred to as *elasticity* [42], [43]. The elasticity with MRE is, like TE, expressed in kPa. Its magnitude is by rule 1/3 of the stiffness value measured with TE [32]. In practice, however, this 1/3 rule does not apply exactly.

For the acquisition of MR elastograms of the liver, mechanical waves of 50 Hz were applied to the liver by a portable transducer, which was placed against the right side of the chest. MRE was performed on a 3.0 Tesla MR system (Intera, Philips Healthcare, The Netherlands). Transverse slices were obtained through the mid-level (feet-head direction) of the liver using a motion sensitive 2D spin-echo based echo-planar imaging sequence with the following acquisition parameters: repetition time 560 ms, echo time 40 ms, FA 90°, number of slices 7, slice thickness 4 mm; field of view 320×320 mm and matrix size 80×80. MRE acquisition time was 78 seconds, divided over 6 breath holds on expiration. Elastograms of the liver were reconstructed using dedicated post-processing software (provided by R. Sinkus, Clichy, France) [43]. A region of interest was manually drawn in the right liver lobe, avoiding liver margins and major blood vessels (Figure 2). Elasticity measured inside this region of interest was used for analysis.

### Laboratory Parameters of GD Severity, Iron Metabolism and Hemochromatosis Mutation Analysis (GD Patients Only)

Concentrations of chitotriosidase, angiotensin-converting enzyme (ACE), ferritin, plasma iron, total iron binding capacity (TIBC), iron saturation, and transferrin and hemoglobin are determined regularly as standard-of-care in our GD patients. Chitotriosidase activity was measured using the 4MU-deoxychitobioside substrate, as described by Aguilera et al. and slightly modified by Schoonhoven et al [44], [45]. Genotyping for the chitotriosidase 24-bp duplication was performed by PCR [46]. Chitotriosidase values of patients who were heterozygous for this chitotriosidase mutation were multiplied by 2. Both ACE and Ferritin were measured as markers of GD activity. Plasma iron, TIBC, iron saturation and transferrin were included to assess iron metabolism.

HFE mutations are relatively common in the Northern European population; 3.5–15% is heterozygous for the C282Y mutation and 20% is heterozygous for the H63D mutation [29], [47]. As high ferritin levels are a common feature in both GD and hemochromatosis, it has been suggested that the possibility of concurrent genetic hemochromatosis should be excluded in GD patients [48]. Therefore all GD patients in this study were tested for the common C282Y and H63D mutations in the HFE gene to investigate the presence or absence of hereditary hemochromatosis/carriership.

### Statistical Analysis

For quantitative imaging, results of non-Sx GD patients were compared with those of the matched controls. Comparisons between non-Sx and Sx GD patients were made for all study parameters. Non-parametric, two-tailed tests were employed on all statistical analyses. For correlations, Spearman’s rho was calculated. For comparison of means and proportions, Mann Whitney U and Fisher’s exact test was used. A p-value of less than 0.05 was considered statistically significant. All analyses were performed with PASW statistics 18 (SPSS, Chicago, Ill, USA) and GraphPad Prism 5 (GraphPad Software, INC, La Jolla, CA, USA).

## Results

### Participants

Of the 23 included participants (16 GD and 7 controls), 21 completed all study procedures. Two patients were excluded after inclusion: one Sx GD patient was unable to participate due to complications unrelated to the study procedures; one non-Sx GD patient withdrew consent before study procedures were completed. One patient was included shortly after undergoing a partial liver resection of segment IV because of a HCC.

MR-LIC measurement was technically unsuccessful initially in one patient, but was repeated successfully six months later. Due to prolonged technical problems with our TE system, examinations could not be performed in six participants during the visit in which MRI was performed and biochemical data were collected. The TE examinations were completed three to twelve months later.

Anthropometric data of all participants and disease characteristics of GD patients, including age at Sx, age at start of treatment, history of iron supplementation and/or blood transfusions and GD genotype, are summarized in Table 1 and 2.

**Table 1:** Participant characteristics.

	Controls (*n* = 7) median (range)	Non-Sx GD (*n* = 7) median (range)	Sx GD (*n* = 7) median (range)
**Sex (m/f)**	6/1	6/1	4/3
**Age (y)**	44 (26–59)	46 (24–61)	55 (39–70)
**BMI (kg/m2)**	22.7 (20.8–28.6)	23.1 (21.1–27.4)	24.8 (21.0–27.7)
**Age Sx (y)**			20 (4–32)
**Age start treatment (y)**		37 (17–45)	43 (29–59)
**Treatment (ERT:SRT)**		6∶1	6∶1
**Genotype**			
N370S/N370S		0	1
N370S/L444P		4	2
N370S/other or unknown		3	4

**Table 2:** Iron status GD patients.

Case no	Age	Sx*	HFE genotype	Blood transfusions	Iron suplementation	LIC (SD) (Gandon)	TE (kPa)
1	43	No	wt/wt	Yes, 5–6 age 8; donor age 19–21	Yes, not in the last 12 years	25 (20)	4.4
2	46	No	wt/wt	No	Yes, briefly age 10	60 (30)	4.8
3	43	No	wt/wt	No, donor age 17–20	No	40 (20)	6.1
4	24	No	wt/wt	No	No	35 (20)	6.6
5	61	No	H63D/H63D	No	Yes, age 15–20	30 (20)	7.2
6	49	No	C282Y/wt	No, donor age 25–29	Yes, age 15	**90 (30)**	3.4
7	59	No	wt/wt	No, donor age 25–40	No	20 (20)	4.2
8	39	Yes	wt/wt	Yes, 1× age 20	Yes, several months, not recently	28 (20)	7.2
9	55	Yes	H63D/H63D	Yes, >40× until age 36	Yes, childh./adol.	**95 (30)**	3.5
10	48	Yes	H63D/wt	Yes, 5–10× childhood	Yes, childh./adol.,briefly age 41	35 (20)	10.5
11	70	Yes	H63D/wt	Yes, >80× age 48, 1× age 56	Yes, childhood	10 (20)	8.7
12	66	Yes	C282Y/wt	Yes, 4× age 17–50	Yes, several days age 17–50	**170 (50)**	9.9
13	55	Yes	H63D/wt	Yes, 1× age 26	Yes, age 15–16	30 (20)	8.8
14	63	Yes	H63D/wt	Yes, 40 units until age 45	No	**250 (50)**	16

* Sx = splenectomy.

### Quantitative Imaging (All Participants)

#### Liver iron concentration

**Figure 3:**
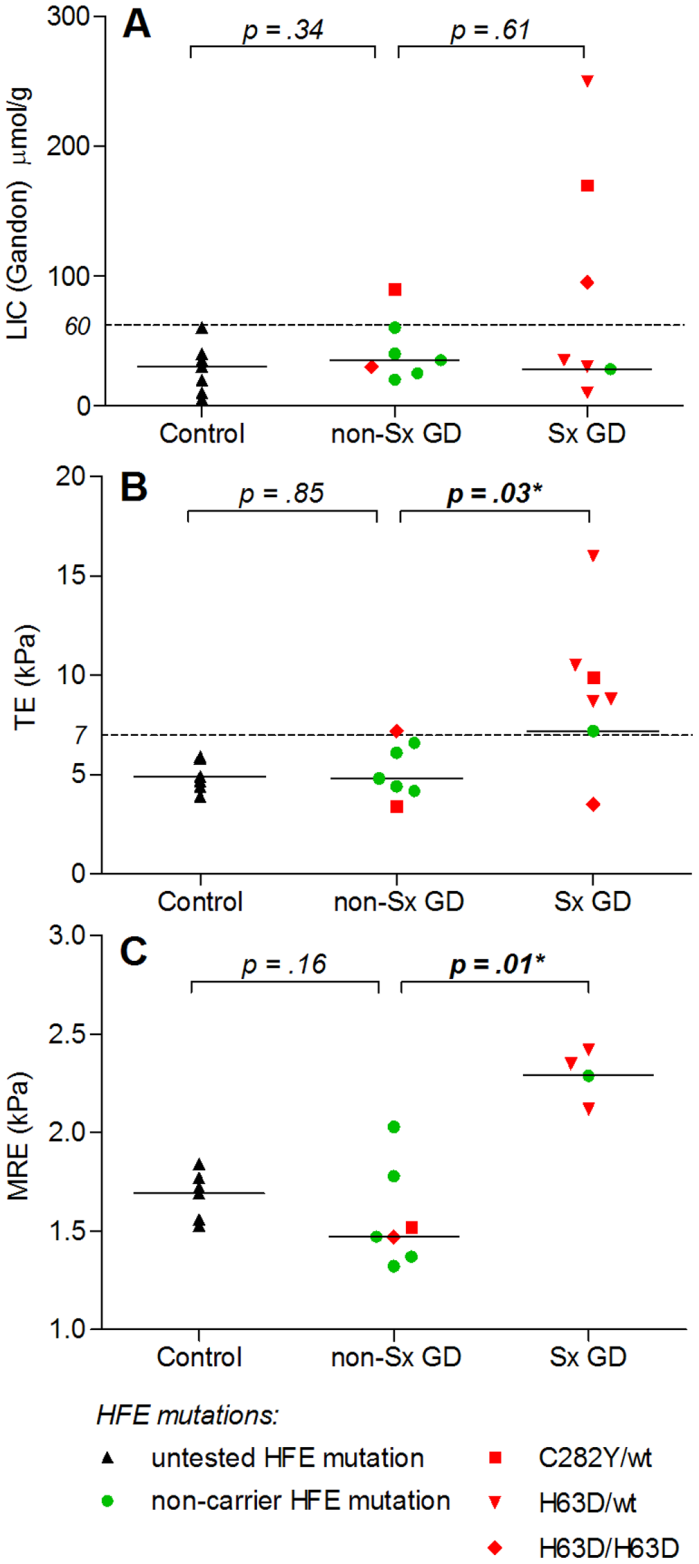
Comparison of quantitative imaging results and distribution of HFE mutations. Solid black horizontal lines represent median values. Presence and distribution of HFE mutations in GD patients are shown in red and green. (A) MR-liver iron concentration (Gandon). Dashed line represents upper limit of normal (60 µmol/g). Median (range) in µmol/g: controls (*n = 7*) 30 (5–66); non-Sx GD (*n = 7*) 35 (20–90); Sx GD (*n = 7*) 35 (10–250). (B) Transient elastography results. Dashed line represents cutoff value for the presence of substantial liver fibrosis (Metavir ≥ F2). Median (range) in kPa: controls (*n = 7*) 4.9 (3.9–5.9); non-Sx GD (*n = 7*) 4.8 (3.4–7.2); Sx GD (*n = 7*) 8.8 (3.5–16.0). (C) MR elastography results. Median (range) in kPa: controls (*n = 7*) 1.69 (1.53–1.84); non-Sx GD (*n = 7*) 1.47 (1.32–2.03); Sx GD (*n = 4*) 2.32 (2.12–2.42).

**Figure 4:**
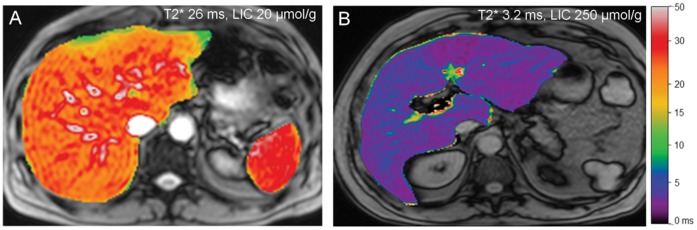
T2* maps of the liver. (A) Healthy control with a normal liver T2* value of 26 ms (LIC-Gandon: 20±20 µmol/g). (B) Sx-GD patient with severe iron overload: T2* value in the liver is short: 3.2 ms; the corresponding LIC measured with the Gandon method was high: 250±50 µmol/g.

LIC values calculated using the method of Gandon showed excellent correlation with T2* measurements (rho = 0.85, *p*<0.01). LIC was elevated (>60±30 µmol/g with the Gandon method) in four GD patients of whom 3 were splenectomized. There were no significant differences between non-Sx GD and Sx GD patients, or between non-Sx and controls (Figure 3 A, Table 3). Reconstruction of T2* anatomical maps of the liver (Figure 4) visualizes distinct differences in LIC between participants.

**Table 3:** Quantitative imaging results.

	Controls (*n* = 7)median (range)	Non-Sx GD (*n* = 7)median (range)	Sx GD (*n* = 7)median (range)	*p*	
				Controls vs. non-Sx	Non-Sx vs. Sx
**LIC-Gandon** **(µmol/g)** ref <60±30 µmol/g	30 (5–60)	35 (20–90)	35 (10–250)	0.34	0.61
LIC T2* (ms) ref >22 ms	26 (16–33)	19.0 (8.7–27.0)	23.0 (3.2–32.0)	0.07	0.95
**TE (kPa)** ref <7.0 kPa for<F2	4.9 (3.9–5.9)	4.8 (3.4–7.2)	8.8 (3.5–16.0)	0.85	**0.03**
**MR Elastography (kPa)** ref unknown	1.69 (1.53–1.84)	1.47 (1.32–2.03)	2.32 (2.12–2.42); *n* = 4	0.16	**0.01**

#### Transient Elastography

TE measurements of both the anterior and posterior locations were difficult to perform, with very low success rates. Results are therefore only reported for the reference location. Of note is that TE results at the reference locations were *poorly reliable* according to the criteria by Boursier et al [35]: in two participants: one non-Sx GD patient (TE 7.2 kPa, IQR 35%) and one Sx GD patient (TE 16.0 kPa, IQR 64%). For the latter patient, liver cirrhosis was also confirmed histologically. Using a cutoff of 7.0 kPa for the presence of substantial fibrosis (stage F2 or higher) with TE, seven GD patients would be classified as having substantial fibrosis (1 non-Sx, 6 Sx), versus none of the healthy controls [43].

Sx GD patients had significantly higher stiffness values compared with non-Sx GD patients (p = 0.03), as shown in Figure 3 B and Table 3. There were no significant differences between non-Sx and controls**.**

#### MR elastography

In three Sx GD patients, acquisition of MRE was not possible due to high liver iron concentrations, causing complete MR signal loss. The four successful MRE acquisitions in the Sx GD group were significantly higher than the non-Sx GD group (p = 0.01), as shown in Figure 3 C. Again, there were no significant differences between non-Sx and controls (Table 3).

### Laboratory Parameters of GD Severity, Iron Metabolism and Hemochromatosis Mutation Analysis (GD Patients Only)

Sx GD patients had significantly higher ACE concentrations (*p*<0.01) compared with non-Sx GD patients. The difference in chitotriosidase activity showed a trend (*p* = 0.07). There was no significant difference in ferritin concentration or any of the other parameters of iron metabolism (Table 4). Although 9 of 14 GD patients had an elevated ferritin concentration, only one Sx patient showed signs of increased iron storage, which is defined as a ferritin concentration above the upper limit of normal combined with an iron saturation >45% [44]. This patient was heterozygous for the H63D mutation.

**Table 4:** Biochemical assessment.

	Non-Sx GD (*n* = 7)median (range)	Sx GD (*n* = 7)median (range)	*p*
Chitotriosidase activity (nmol/ml.h) *ref 10–190 nmol/ml.h*	3,318 (1,269–16,177)	9,160 (4,175–31,021); *n* = 6	0.07
**ACE (U/L)** *ref 20–70* *U/L*	65 (53–100)	124 (85–150); *n* = 6	**<0.01**
**Hemoglobin level (mmol/L)** *ref ♂ 8.5–10.5 mmol/l ♀7.5–10 mmol/L*	9.4 (8.8–9.8)	8.4 (6.8–9.2)	**<0.01**
**Ferritin (µg/L)** *ref ♂ 25–300 µg/L, ♂premenopausal 15–200, ♀ postmenopausal 20–250*	315 (85–802)	475 (219–3,109)	0.13
**Transferrin (g/L)** *ref 2–3.6 g/L*	2.22 (1.95–2.72)	2.05 (1.72–3.43)	0.70
**Iron (µmol/L)** *ref ♂ 11–32 µmol/L, ♀ 11–27 µmol/L*	15.6 (11.9–16.1)	21.6 (10.6–30.9)	0.16
**TIBC (µmol/L)** *ref 50–91 µmol/L*	55.9 (49.1–68.5)	51.7 (43.3–86.4)	0.70
**Iron saturation** *ref 0.20–0.55*	0.25 (0.22–0.31)	0.31 (0.24–0.65)	0.10

HFE mutations were present in 8 of 14 GD patients (57%): two C282Y heterozygotes, two H63D homozygotes and four H63D heterozygotes. Six out of 7 Sx GD patients and two out of 7 non-Sx GD patients carried a mutation (*p* = 0.10). Figures 3 A–C show the distribution of HFE mutations between Sx and non-Sx groups in relation to LIC-Gandon, TE and MRE.

### Relationships between Study Parameters

#### Liver iron concentration

**Figure 5:**
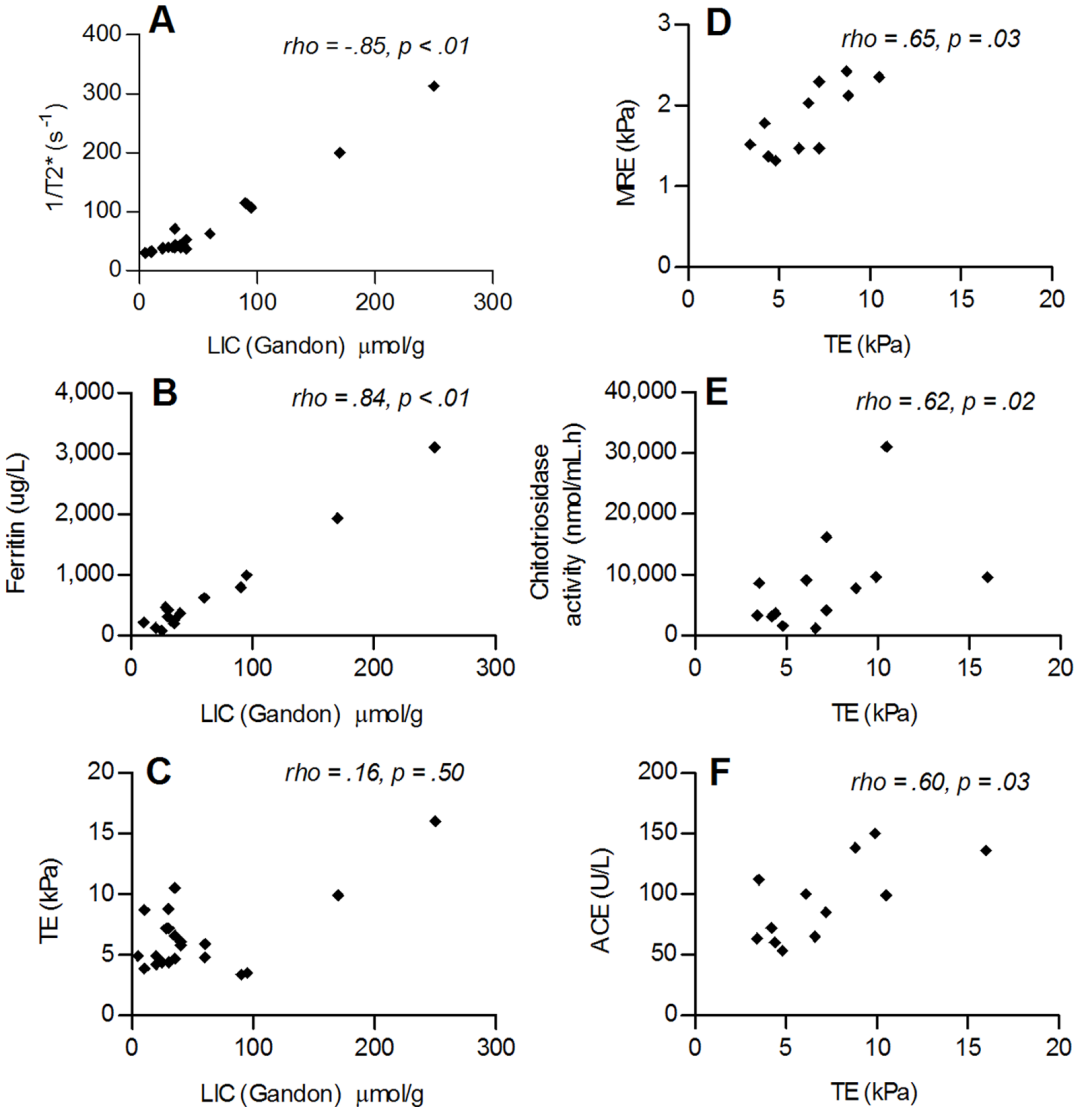
Correlations between study parameters. A–C: correlations between MR-LIC and 1/T2* (*n = 21*), TE (*n = 21)* and ferritin concentration (*n = 14*). D–F: correlations between TE and MRE (*n = 18*), Chitotriosidase activity (*n = 14*) and ACE (*n = 14)*.

Besides a significant correlation between LIC-Gandon and T2* (rho = 0.85, p<0.01), we observed a strong correlation between LIC-Gandon and ferritin concentration (rho = 0.84, p<0.01), and between LIC-Gandon and transferrin concentration (rho = −0.62, p = 0.02), Figure 5 A–C. LIC-Gandon did not correlate with the remaining biochemical parameters, or with TE (rho = 0.10, p = 0.73) or MRE (rho = −0.40, p = 0.23). Correlations of T2* with the aforementioned parameters were similar to those observed for LIC-Gandon (data not shown).

Of 4 patients with elevated LIC, the 3 splenectomized patients (no 9, 12, 14, table 2) had received blood transfusion in their past, although the number of transfusions varied. The fourth, non-splenectomized patient (no 6) had not received blood transfusions, but was treated with iron supplementation for a few months as an adolescent. Conversely, 3 patients with a history of multiple blood transfusions (no 1, 10, 11) had normal LIC.

#### Transient elastography

TE measurements correlated significantly with MRE (rho = 0.67, p = 0.02), with chitotriosidase activity (rho = 0.62, p = 0.02) and with ACE (rho = 0.60, p = 0.03), Figure 5 D–F. Correlations between TE measurements and plasma iron concentration (rho = 0.50, p 0.07) and iron saturation (rho = 0.47, p 0.09) showed a trend. There was no significant correlation with ferritin, total iron binding capacity or transferrin.

## Discussion

This study is the first to demonstrate that liver stiffness values are significantly higher in splenectomized GD patients when compared with GD patients with an intact spleen, as measured by TE and MRE. TE and MRE techniques can be used to identify and monitor GD patients to assess the presence of liver fibrosis. We also showed that liver stiffness parameters and liver iron content in non-Sx GD patients were not different from their matched healthy controls. Considering our hypothesis that splenectomized patients are particularly at risk for the development of HCC, we suggest that this risk is related to the presence of fibrosis in the liver.

We did not add a splenectomized control group as fibrosis is not caused by splenectomy, but rather splenectomy indicates advanced Gaucher disease. A similar relationship between risk for bone complications and splenectomy has previously been shown [23], [49], [50].

One patient was included shortly after undergoing a resection of a liver segment because of HCC. He had a history of severe GD including a splenectomy, bone complications and anemia despite splenectomy, requiring transfusions (approximately 40 units of packed red blood cells) before the start of enzyme replacement therapy (ERT). PCR testing for hepatitis B and C was negative in this patient. Interestingly, we found this patient to have both the highest concentration of liver iron (250±50 µmol/g), as well as the highest liver stiffness value with TE: 16.0 kPa, a value being suggestive of the presence of severe liver fibrosis and probable cirrhosis [51]. Histopathological analysis of resected liver parenchyma that surrounded the tumor confirmed both liver cirrhosis and severe iron overload. HFE mutation analysis showed heterozygosity for the H63D mutation. MRE was unsuccessful due to the high iron load. This patient started ERT 18 years ago with quick and complete recovery of blood counts but with remaining high chitotriosidase activities, indicative of residual Gaucher cell burden.

Although not previously used in GD, numerous studies have demonstrated the ability of TE to predict liver fibrosis, cirrhosis and cirrhosis-related complications in various chronic liver diseases [31], [51], [52]. The European Association for the study of the Liver (EASL) have recently included TE in their guidelines for assessing disease severity in hepatitis C [53]. MRE is still under development and is at this point mainly used as a research tool. The technique also has a good diagnostic accuracy for non-invasive fibrosis assessment [54]. A head-to-head study comparing the accuracies of MRE and TE was in favor of MRE [32]. However, the fact that MRE measurements were unsuccessful in three of seven Sx GD patients due to high liver iron load – while TE was successful - highlights an important limitation of this technique at present. TE identified high stiffness of the liver in two of these three cases, which suggests that these patients were at risk, and illustrates that this subgroup should not be missed. A technical solution for this limitation of MRE is already being developed, making use of short echo times and fractional encoding [42].

This study also revealed a difference in the frequency of mutations in the HFE gene between splenectomized and non-splenectomized patients. This difference was found by chance, since screening was performed primarily to exclude hereditary hemochromatosis as a cause for iron accumulation. The majority (6 of 8) involved H63D mutations, which have not been associated with clinically significant iron storage, unless present in combination with the C282Y mutation [55]. Although this finding might be suggestive of a relationship between mutations in the HFE gene and GD, further evaluation - in a larger cohort - is warranted regarding the relationship between HFE mutations and other parameters of GD severity.

Stein et al investigated the occurrence of iron overload in GD type I patients and found signs of iron overload in only 3 of 114 patients, 2 of whom carried the H63D mutation (1 homozygous, 1 heterozygous) in de HFE gene - as compared with 38/114 in the total cohort [48]. In contrast, our study revealed signs of iron overload, albeit to varying extent, in 4 of 14 GD patients. This difference might be explained by the difference in the populations that were studied: 53% of the population studied by Stein et al. was homozygous for the N370S mutation, which is generally associated with milder disease manifestations, whereas we had only one such patient in our cohort.

We did not find a relationship between LIC and fibrosis in our study. First of all, patients with increased liver stiffness did not always have an elevated LIC, suggesting that other factors than iron storage must be involved as well. Four patients had iron overload (>60±30 µmol/g), of whom two had moderately elevated LIC values of 170±50 and 250±50 µmol/g, and liver stiffness values indicating the presence of substantial fibrosis: 9.9 and 16.0 kPa, respectively (TE). In the other two patients, with mildly elevated LIC values of 90 and 95 µmol/g, TE values were normal. Bassett et al described fibrosis and cirrhosis in patients with hereditary hemochromatosis only at hepatic iron concentrations exceeding 400 µmol/g [56]. Guyader et al did not confirm these results, but in their cohort comprising 197 patients with homozygous C282Y hemochromatosis, median LIC of patients with no or moderate fibrosis (F0–F2) was 250 µmol/g versus 461 µmol/g in those with severe fibrosis and cirrhosis (F3–F4) [57]. The previously stated example of our patient who developed HCC further illustrates the complexity of an analysis of the relationship between LIC and fibrosis, which may be confounded by HFE mutation status and history of transfusions. Nevertheless, HCC risk in GD patients is perhaps best explained as the result of a combination of local effects of GD storage cells and these (GD associated) factors.

Based on the MRI technique used, we are unable to differentiate between iron stored in Gaucher cells and iron stored in hepatocytes. The pattern of iron deposition may influence toxicity. While iron deposits have been described in Gaucher cells, parenchymal iron storage has been observed in GD patients who developed HCC as well (personal observations).

A limitation of this study is that we did not perform laboratory assessments of iron storage and screening for HFE mutations in our control group, since we wanted to focus on the novel imaging techniques and limit the invasiveness of participation in this study. In the GD groups, biochemical parameters were assessed for routine clinical practice, so only additional consent had to be given for the HFE mutation screening. However, none of the controls had signs of hemochromatosis based upon LIC data.

Furthermore, the high IQRs associated with some of the liver stiffness measurements using TE. A common recommendation for validation of successful measurements is that the interquartile range should be less than 30% of the median liver stiffness measurement value. A study by Lucidarme et al identified IQR as a factor of overestimation of liver fibrosis and suggested an even stricter cutoff value of 0.21 [58]. Castera et al identified an operator experience fewer than 500 examinations as a reason for unreliable results, which is a factor that may have influenced the current study results [31]. However, repeating TE analyses while excluding the three participants in whom IQR exceeded 30% did not change statistical significance (data not shown).

All GD patients were treated with either enzyme replacement therapy or substrate reduction therapy. Whether this has influenced the parameters of liver stiffness or iron content cannot be concluded from this study. The differences between splenectomized and non-splenectomized GD patients and the observation that splenectomy can be avoided by ERT suggests that ERT may decrease the risk for development of hepatic fibrosis and cirrhosis- related complications in the future.

In conclusion, TE and MRE can be used to detect and monitor liver fibrosis in patients with GD. We hypothesize a relationship with excessive hepatic iron accumulation in splenectomized patients. However, the role of iron storage needs further study. Since the splenectomized GD group showed more advanced liver disease than the non-splenectomized GD group, we recommend that all splenectomized patients, especially those with evidence of substantial liver fibrosis undergo regular screening for HCC, according to current guidelines.
